# Screening for Structural Hemoglobin Variants in Bahia, Brazil

**DOI:** 10.3390/ijerph13020225

**Published:** 2016-02-18

**Authors:** Wellington Santos Silva, Roberto Ferreira de Oliveira, Sanzia Bezerra Ribeiro, Isabel Batista da Silva, Edna Maria de Araújo, Abrahão Fontes Baptista

**Affiliations:** 1 Bahia Adventist College, 44300-000 Cachoeira, Bahia, Brazil; prof.robertoferreira@icloud.com (R.F.O.); sanziar@gmail.com (S.B.R.); isabelambrosio-enf@hotmail.com (I.B.S.); 2 Functional Electrostimulation Laboratory, Biomorphology Department, Health Sciences Instituto, Federal University of Bahia, Av. Reitor Miguel Calmon, s/n Vale do Canela, 40110-902 Salvador, Bahia, Brazil; abrahao.baptista@gmail.com; 3 Graduate Program on Medicine and Health, Faculty of Medicine, Federal University of Bahia, Rua Augusto Viana s/n Canela—Hospital Universitário Prof. Edgard Santos, 5o andar, 40110-060 Salvador, Bahia, Brazil; 4 Feira de Santana State University, Health Department, 44031-460 Feira de Santana, Bahia, Brazil; ednakam@gmail.com

**Keywords:** hemoglobin variants, sickle cell disease, newborn screening

## Abstract

Brazil was the country that received the largest number of Africans during the time of colonization, and Bahia was the Brazilian state that received the largest number of slaves from Africa. The purpose of this study was to evaluate the coverage of the newborn screening program for sickle cell disease in the Recôncavo Baiano region of the state of Bahia, and to show the frequency of the subjects with hemoglobin variants in the 2006–2009 period. Blood samples from neonates in twelve cities in the Recôncavo Baiano region were analyzed by High Performance Liquid Chromatography. A total of 16,402 children were born in this period, 14,773 of which underwent newborn screening. In this period 1416 children were born carrying hemoglobin variants HbS and HbC. Forty-seven patients—20 HbSS genotype and 27 HbSC genotype—were diagnosed in eleven of the twelve cities surveyed. The proportion of children born with sickle cell disease in the Recôncavo Baiano region was 1/314, which was higher than the 1/650 rate for the state of Bahia. The data presented in this study confirm the high frequency of sickle cell disease in Recôncavo Baiano, demonstrating the need to create a referral center for the care of patients with sickle cell diseases in the region.

## 1. Introduction

Sickle cell diseases are a group of inherited diseases, the cause of which is the presence of hemoglobin S from a point mutation in the β-globin gene, leading to a glutamic acid substitution of the amino acid valine at the sixth position of the β chains. All genotypes include at least one copy of the β^S^ allele in combination with one or more mutations in the β-globin gene [1]. Sickle cell anemia (HbSS) is usually responsible for most cases of sickle cell disease, with most of the remainder having hemoglobin SC disease (HbSC disease) owing to the inheritance of the β^S^ and β^C^ alleles [2].

In Brazil, the introduction of hemoglobin S occurred through the slave trade of many African tribes were brought to the country to perform slave labor in the sugar cane industry in the Northeast and later for the extraction of precious metals in Minas Gerais [3,4].

The first newborn screening programs were implemented in some countries in the 1970s and 1980s [5]. Newborn screening for sickle cell diseases is important for the early treatment of these diseases, which pose a public health problem in Brazil [6]. Government Ruling MS 822/01 by the Brazilian Ministry of Health made newborn screening mandatory for hemoglobinopathies in 2001, with a special focus on sickle cell disease [7].

Early diagnosis has shown a significant impact on the morbidity and mortality of patients with sickle cell disease because it allows the early introduction of the affected newborns into specific health care programs. It allows education of the parents on the identification of both the early signs and the symptoms of the complications that indicate prophylaxis against pneumococcal infections, as well as the determination of the risk of other serious complications and the performance of genetic counseling [8].

The distribution of the hemoglobin S gene in Brazil is very heterogeneous, depending on the composition of the Negroid and Caucasoid populations in the different regions of the country. Thus, the prevalence of the sickle cell trait (HbAS) is higher in the Northern and Northeast regions (6% to 10%), while in the Southern and Southeast regions the prevalence is lower (2% to 3%). There are more than two million Brazilian carriers of the sickle gene and more than 30,000 individuals with Hb SS. It is estimated that one child is born with sickle cell diseases for every thousand live births in the country. The incidence of sickle cell disease varies among newborns in the Brazilian states, from 1:650 in the state of Bahia in the Northeast to 1:13,000 in the state of Rio Grande do Sul in the South [9]. 

Bahia is the state with the highest frequency of sickle cell diseases in Brazil. Recôncavo Baiano, the focus of our study, is the region surrounding a large bay on the Atlantic Coast of Brazil, bordered to the north by the state capital of Salvador. This region, which includes many historic and economically important cities, has had a long, close association with the state capital and with the history of the African slave trade. As a result, Recôncavo Baiano has many Afro-derived Brazilian sub-populations or “quilombos”, which were originally founded by runaway slaves [10].

In the state of Bahia, newborn screening for sickle cell disease started in June 2000 through the initiative of the Parents and Friends of Children with Mental Diseases Association of Salvador (Associação de Pais e Amigos dos Excepcionais—APAE-Salvador) in partnership with the Health Department in the state of Bahia. In 2007, the Newborn Screening Program of Bahia managed to reach 417 cities of the state, strengthening the partnership between the three levels of government, which are represented by the Ministry of Health, Department of Health and the Health Departments in the cities and the APAE Research and Diagnostic Center [11].

In the state of Bahia, screening for sickle cell disease is part of the newborn screening program that includes screening for phenylketonuria and congenital hypothyroidism. The test is free of charge for all children when the mother takes the child to the first vaccine at the health unit.

Newborns diagnosed with hemoglobin variants are called upon to perform confirmatory tests through the active search service of APAE Research and Diagnostic Center. The active search service contacts the health unit of cities to immediately locate the newborn with suspected sickle cell disease or sickle cell trait. The goal is to bring this child to confirm the diagnosis, introduction of treatment of confirmed cases of sickle cell disease and genetic counseling for parents.

The first study on the newborn screening coverage for sickle cell diseases in Recôncavo Baiano revealed that the number of live births that had received the heel prick test was still deficient. Another study in two other cities in Recôncavo Baiano revealed a high frequency of sickle cell diseases. Due to the natural history of the settlement of Recôncavo Baiano, this region varies in the prevalence of sickle cell diseases, but its frequency is high in many cities [12,13].

The objective of this study was to assess the newborn screening coverage for sickle cell diseases in 12 cities located in the Recôncavo Baiano region during the 2006–2009 period and to show the prevalence of the carriers of the hemoglobin variants Hemoglobin HbS, HbC and HbD in those cities that do not yet have a referral service for the treatment of these patients.

## 2. Materials and Methods

To evaluate the coverage of the newborn screening for the hemoglobinopathies program, we obtained information on the number of infants who participated in the newborn screening at the APAE Research and Diagnostic Center of Salvador through technical High Pressure Liquid Chromatography (HPLC) as described previously [14,15] using the Sickle Cell^®^ kit and the machine Variant Hemoglobin Testing System (Bio-Rad Laboratories Inc.: Hercules, CA, USA). The results of hemoglobin variants were confirmed by isoelectric focusing (IEF, PerkinElmer: Waltham, MA, USA) in the 2006–2009 period in 12 cities in the Recôncavo Baiano of which 10 are part of the 31st DIRES (Regional Health Division), one of the 1st and the other of the 4th. The APAE Research and Diagnostic Center is the only accredited by the Ministry of Health and the State Department of Health, to perform neonatal screening in the state of Bahia. It is certified by the following quality control centers: Centers for Disease Control and Prevention/CDC Atlanta, USA, the International Organization for Standardization (ISO 9001), and Brazilian Society of Quality Control of Clinical Pathology and Laboratory Medicine.

The number of births in this region was obtained from the Brazilian National System of Live Births (SINASC) and the System of Primary Care Information (SIAB) through the website of the Secretariat of the State of Bahia Health (SESAB). This project was approved by the Ethics Committee of the State University of Feira de Santana (protocol number 0075.059.000-11).

## 3. Results

During the period of 2006 to 2009, of the 16,402 children who were born, 14,773 underwent newborn screening in 12 cities in the Recôncavo Baiano region (there were only 11 cities in the period from 2006 to 2008, until the city of Governador Mangabeira was included in 2009). The newborn screening coverage ranged from 85.5% in 2006 to 94.2% in 2009. The town of Conceição do Almeida had the lowest coverage in the period, with 29.3% in the year 2007. The city of Cruz das Almas had the highest coverage for the region (Table 1).

The frequency of the newborns with sickle cell trait (HbAS genotype) in 2006 ranged from 2.6% in the city of Sapeaçu to 8.7% in the town of Conceição do Almeida, with an average of 6.2% for the region. In 2007, the rate was 6.3% for the region, with Cachoeira being the city with the highest rate (9.2%). In 2008 and 2009, the frequency of the newborns with sickle cell trait in the region was 5.5% (Table 2).

The frequency of the newborns with HbAC genotype ranged from zero in 2007 in the city of Muritiba to 6.6% in 2008 in the city of Cabaceiras do Paraguaçu. The total estimated frequency of the carriers for the two hemoglobin variants in these populations was 10.4% in 2006, 9.8% in 2007, 9.5% in 2008 and 8.9% in 2009. For the entire period, the frequency was 9.6% (Table 2). Four newborns with HbAD genotype were also found, corresponding to a frequency of less than 0.03%. Homozygotes for the hemoglobins HbC and HbD were not found.

In 2006, nine newborns were diagnosed with sickle cell diseases, two with sickle cell anemia (HbSS genotype), and seven with HbSC genotype in the cities of Cachoeira, Cruz das Almas, Muritiba and Maragogipe. In 2007, seven patients with HbSS genotype and seven with HbSC genotype were diagnosed in the cities of Cabaceiras Paraguaçu, Cachoeira, Cruz das Almas, Muritiba, São Felix and Sapeaçu. In 2008, four patients with HbSS genotype were diagnosed in the cities of Cachoeira, Cruz das Almas, and São Felipe and Saubara. In the same year, five newborns with HbSC genotype were diagnosed in Cabaceiras do Paraguaçu and Cruz das Almas. In 2009, 16 more patients were diagnosed in seven cities. A total of 20 newborns with HbSS genotype and 27 with HbSC genotype were diagnosed in the period, corresponding to the frequencies of 0.13% and 0.18%, respectively (Table 3). 

Table 4 shows the proportion of infants with sickle cell diseases in the cities studied. In particular, in the city of Cachoeira, 0.7% of children were born with sickle cell disease. The proportion of cases with sickle cell disease was 1/134 births in the county.

## 4. Discussion

The number of newborns who were screened in some cities was incongruent with the number of births (Table 1). This discrepancy is observed because some children underwent the test more than once in different cities, leading to this anomaly.

Comparing the results about the newborns coverage in the 2006–2009 period with a previous study to assess the coverage of the newborn screening in the cities of Cachoeira, Maragogipe and São Felix [12], we observed an increase in the coverage in the cities of Cachoeira and Maragogipe. Nevertheless, some cities, such as Muritiba and Conceição do Almeida presented estimates below half of the number of newborns in the period 2006–2009. Although the APAE Research and Diagnostic Center offers training and materials, some cities are not fully prepared to carry out the specific data collection.

The number of sickle cell trait AS carriers for this population was one to every 17 individuals. Considering the number of heterozygotes for HbS and HbC hemoglobin, the number rises to one in every 10 individuals. This number is higher than in the rest of the state of Bahia. The data show that 1% of couples of reproductive age living in this region have hemoglobin variants HbS and HbC. Because of the low socioeconomic status of this population, predominantly African descent, many heterozygotic couples do not know their genetic condition and are not receiving genetic counseling. In Brazil, due to public health policies, genetic counseling aims to inform couples about the disease and treat patients, leaving the decision to have children or not, up to the parents.

It is puzzling that sickle cell trait HbAS carriers were consistently more frequent than AC carriers in the populations of newborns screened and the number of SS genotype was smaller than the number of SC genotype. According to the observed, the number of genotypes in the population is not in accordance with the Hardy-Weinberg equilibrium (χ^2^ = 16,532; *fd* = 3; *p* < 0.05). The test to evaluate the excess of heterozygotes was performed using the GDA software [16] and the result was not significant (*p* = 0.56). The inbreeding coefficient did not reveal excess of homozygotes (*fis* = 0.0038). Therefore, these results do not indicate differential survival of AS heterozygotes or endogamy. The observed deviations from the Hard-Weinberg equilibrium for the genotype frequencies can happen by chance.

In this study patients were diagnosed in 11 of the 12 cities surveyed. The proportion of children born with sickle cell disease in Recôncavo Baiano is larger than in the rest of the state of Bahia. While the proportion of cases in the state is 1/650 births, the region of the Recôncavo Baiano region has a case rate of 1/314 births. To get an idea of the magnitude of these results we can compare with the published data for the USA population. During the 20-year period (1991–2010), there were 39,422 confirmed cases of sickle cell disease among 76,527,627 newborn births screened (1:1941) and 1,107,875 laboratory reports of probable sickle trait among 73,951,175 newborn births screened (1:67). The highest sickle cell disease and sickle cell trait incidence during the 20 years was in the District of Columbia (1:437 and 1:22 respectively) [17].

These results place the Recôncavo Baiano as one of the regions with the highest frequency of hemoglobin variants in the American continent, and the newborn screening coverage in some of this region’s cities is still a challenge. The pressure from the black social movement in Brazil led to inclusion of sickle cell diseases and other hemoglobinopathies in the national neonatal screening, and the consequent creation and institutionalization of the National Neonatal Screening Program (NNSP) in 2001. This action preceded the creation of the National Integral Attention Policy to people with sickle cell diseases in 2005 and the National Integral Health of the Black Population Policy (NIHBPP) in 2009 by the Brazilian Health Ministry. Although, despite the fact that sickle cell anemia is defined by NIHBPP as the most prevalent genetically determined disease, actions under this policy, to reduce their prevalence, have proved timid, nascent or nonexistent in most of Brazilian municipalities. In contrast, despite its limitations, NNSP has proven to be the greatest advance in the early detection of sickle cell disease, in order to prevent and reduce morbidity and mortality.

## 5. Conclusions

Bahia is the Brazilian state with the highest frequency of patients with hemoglobin variants affecting approximately one in every seventeen people. The frequency of hemoglobin variants HbS and HbC in the Recôncavo Baiano region is higher than the average for the state of Bahia, which is one in every ten individuals. The frequency varies considerably between cities. The proportion of children born with sickle cell disease in the Recôncavo Baiano region was 1/314, which was higher than the 1/650 rate in the state of Bahia. The city of Cachoeira with predominantly African ancestry showed the proportion of cases with one case of sickle cell disease per 134 births in the city. The data presented in this study confirm the high frequency of sickle cell disease in the Recôncavo Baiano region, demonstrating the need to create a referral center with a multidisciplinary team that offers continuing medical and laboratory patient care. This assistance should consist of clinical monitoring of patients and provision of adequate orientation to their families.

## Figures and Tables

**Table 1:** Newborn screening coverage evaluation in Recôncavo Baiano in the 2006–2009 period.

Year	Neonatal Trial	Number Newborns	Coverage (%)
2006	3544	4144	85.5
2007	3472	4012	86.5
2008	3705	3945	93.9
2009	4052	4301	94.2
Total	14,773	16402	90.0

**Table 2:** Frequency of the heterozygotes for the hemoglobin variants in the Recôncavo Baiano region in the 2006–2009 period.

Year	Neonatal Screenning	Number Cases AS	%	Number Cases AC	%
2006	3544	220	6.2	148	4.2
2007	3472	218	6.3	122	3.5
2008	3705	202	5.5	146	4.0
2009	4052	221	5.5	139	3.4
Total	14,773	861	5.8	555	3.8

**Table 3:** Frequency of patients with sickle cell disease in the Recôncavo Baiano region in the 2006–2009 period.

Year	Newborn Screenning	Number Cases SS	%	Number Cases SC	%
2006	3544	2	0.00	6	0.17
2007	3472	7	0.20	7	0.20
2008	3705	4	0.10	5	0.13
2009	4052	7	0.20	9	0.22
Total	14,773	20	0.13	27	0.18

**Table 4:** Proportion of newborns with sickle cell diseases in the cities of the Recôncavo Baiano region in the 2006–2009 period.

City	Number Cases SCD	Number Test	Frequency (%)	Proportion Cases SCD/NBs
Cachoeira	12	1,612	0.7	1/134
Cabaceiras do Paraguaçu	04	990	0.4	1/247
Conceição do Almeida	00	618	0.0	-
Conceição da Feira	02	1,091	0.2	1/515
Cruz das Almas	14	3,810	0.4	1/272
Governador Mangabeira *	03	265	1.1	1/88
Maragogipe	02	2,009	0.0	1/1004
Muritiba	02	789	0.2	1/394
São Felipe	01	1,078	0.0	1/1078
São Félix	02	717	0.3	1/358
Sapeaçu	03	1,168	0.2	1/389
Saubara	02	626	0.3	1/313
Total	47	14,773	0.4	1/314

* Data regarding the collection was available only for 2009.
